# Effectively Sharing Data, Data Visualization and Sharing the Story

**DOI:** 10.1093/geroni/igaf122.1376

**Published:** 2025-12-31

**Authors:** Frances Yang, Katie Krueger, Dan English, Bradley Newell, Teri Kennedy

**Affiliations:** University of Kansas, University of Kansas Medical Center, Kansas, United States; University of Kansas Medical Center, Kansas City, Kansas, United States; University of Kansas Medical Center, Kansas City, Kansas, United States; University of Kansas, Wichita, Kansas, United States; University of Kansas Medical Center, Kansas City, Kansas, United States

## Abstract

This presentation focuses on the sharing of evaluation data in a visual format that diverse stakeholders can utilize to ensure transparency, accountability, and collaboration. Effective data sharing of outcomes is essential for advancing the quality and impact of geriatric education, ensuring transparency, accountability, and collaboration among diverse stakeholders. Through an illustrative case, this presentation highlights the Kansas 4M Geriatric Workforce Enhancement Program (GWEP) Dashboard as an example of optimizing data visualization to drive meaningful insights and improve program outcomes. The dashboard applies FAIR (Findable, Accessible, Interoperable and Reusable) principles to transform complex data into a meaningful story. Using a cloud-based portal, called mySidewalk.com, access to data is streamlined through a keyword-driven search engine, and integrates an AI-powered assistant to support dynamic dashboard creation. Interactive visualizations, including color-coded workforce maps and correlation matrices linking the “4Ms” of Age-Friendly Health Systems – What Matters, Medications, Mentation, and Mobility – enhance interoperability. Additionally, automated saving of searches, templates and reports fosters efficient knowledge sharing on an ongoing basis. By aligning evaluation efforts with strategic data visualization, this dashboard demonstrates how geriatric education initiatives can leverage data to communicate to diverse stakeholders in order to drive sustainability and prioritization of geriatric education, attendees will gain insights into how systematic data-sharing practices can enhance geriatric workforce development and drive investment.

